# Global patterns of workplace productivity for people with depression: absenteeism and presenteeism costs across eight diverse countries

**DOI:** 10.1007/s00127-016-1278-4

**Published:** 2016-09-26

**Authors:** S. Evans-Lacko, M. Knapp

**Affiliations:** 1 Personal Social Services Research Unit, London School of Economics and Political Science, Houghton Street, London, WC2A 2AE UK; 2 Health Service and Population Research Department, Institute of Psychiatry, Psychology and Neuroscience at King’s College London, London, UK

**Keywords:** Mental health, Depression, Employment, Stigma, Productivity

## Abstract

**Purpose:**

Depression is a leading cause of disability worldwide. Research suggests that by far, the greatest contributor to the overall economic impact of depression is loss in productivity; however, there is very little research on the costs of depression outside of Western high-income countries. Thus, this study examines the impact of depression on workplace productivity across eight diverse countries.

**Methods:**

We estimated the extent and costs of depression-related absenteeism and presenteeism in the workplace across eight countries: Brazil, Canada, China, Japan, South Korea, Mexico, South Africa, and the USA. We also examined the individual, workplace, and societal factors associated with lower productivity.

**Results:**

To the best of our knowledge, this is the first study to examine the impact of depression on workplace productivity across a diverse set of countries, in terms of both culture and GDP. Mean annual per person costs for absenteeism were lowest in South Korea at $181 and highest in Japan ($2674). Mean presenteeism costs per person were highest in the USA ($5524) and Brazil ($5788). Costs associated with presenteeism tended to be 5–10 times higher than those associated with absenteeism.

**Conclusions:**

These findings suggest that the impact of depression in the workplace is considerable across all countries, both in absolute monetary terms and in relation to proportion of country GDP. Overall, depression is an issue deserving much greater attention, regardless of a country’s economic development, national income or culture.

## Introduction

According to the most recent Global Burden of Disease statistics, depression ranks as a leading cause of disability worldwide [1], affecting 350 million people [2]. Among all medical conditions, depression may have the greatest negative impact on time management and productivity [3, 4]. In high-income countries, trends suggest that sick days lost to mental health problems such as depression have increased in recent years [5]. In addition to the significant personal consequences associated with depression, the economic impact of these trends can be considerable, including for employers.

In the workplace, depression can influence productivity through increased absenteeism. In addition, depression can influence the performance of workers who are ‘present’ at work, i.e., presenteeism. Previous research suggests that presenteeism accounts for the majority of the costs [6–8]. However, most research has been done in Western, high-income countries, and little is known about how the relationship between depression and workplace productivity varies across countries. Labor market circumstances and culture may influence the relationship between depression and workplace productivity [9]. We (1) estimate workplace productivity (absenteeism and presenteeism) associated with depression across eight diverse countries; (2) make population-level country estimates of annual absenteeism and presenteeism costs associated with depression; and (3) examine individual, workplace and societal factors associated with lower productivity.

## Methods

### Data source

We performed secondary analysis on data collected in the Global IDEA (Impact of Depression in the Workplace in Europe Audit) survey which collected data on presenteeism and absenteeism associated with depression and their correlates. Participants were recruited through an online market research panel. Before joining the panel, participants were screened to: remove duplicates, validate name and surname, validate country based on internet protocol address, validate town and zip/postal code according to official lists, check for valid correlations between sociodemographic data (gender, and age of parents and children), and validate contact information. Individuals, who worked in advertising and/or market research, and those aged under 16 years old were excluded.

Employed people across Brazil, Canada, China, Japan, South Korea, Mexico, South Africa, and the USA were sampled from the online research panels. Selected panel members were invited to participate in the survey through Ipsos MORI (http://www.ipsos-mori.com/) via email. Quotas were set to include equal distributions of age and gender, and the sample was designed to be geographically representative of each country. In addition, as managers were considered of key interest, ten percent of the sample for each country was represented by managers. Response rates varied by country. Reported estimates ranged from around 5 % in China, 8 % in the USA, 10 % in Brazil, Mexico, Canada, and South Africa, 15 % in Japan, and 37 % in South Korea. Questionnaires were collected from approximately 1000 respondents per country.

### Measures

*Sociodemographic information* included age band (18–24, 25–44, and 45–64 years), gender, education level completed (tertiles were created for each country to indicate locally relevant high, medium, and low education categories). Data were collected on annual or monthly household income from individuals in all countries except for China, where *individual*-level income details were collected.

*Previous diagnosis of depression* was determined via self-report by asking respondents: Have you ever personally been diagnosed as having depression by a doctor/medical professional?

### Did not tell employer because of fear of losing job

Employees who reported a previous diagnosis of depression which they did not disclose to their employer were asked whether they did not tell their employer, because they felt it would put their job at risk or in this economic climate they felt that it was too risky.

### Country variables

We used data from the IDEA survey to describe the overall population prevalence of employees with a diagnosis of depression. We derived annual prevalence rates from lifetime prevalence rates based on nationally representative psychiatric epidemiological surveys. Given the standardized cross-country methodology, we used World Mental Health Survey data where available. The ratio of lifetime to annual prevalence of depression ranged from 1.7 in China to 3.0 in Japan. We applied individual country ratios based on data from their own country surveys and also performed sensitivity analyses based on the lowest (1.7) and highest (3.0) ratios from participating study countries. Country unemployment rates for 2013 were taken from the International Labor Organization global employment trends report (World Health Organization 2014). Figures for gross domestic product (GDP) per capita (US $) for each participating country were taken from the World Bank (World Bank [11]).

### Work performance

Self-reported presenteeism was assessed using the WHO Health and Work Performance Questionnaire (HPQ) [12, 13]. For this assessment, respondents rate their overall work performance during the past 4 weeks and this is transformed to a 0–100 scale where 0 corresponds to doing no work at all (while at work) and 100 signifies top work performance. Presenteeism as assessed by the HPQ has been found to be valid when, for example, compared to independent employer records of job performance and supervisor ratings [14]. Absenteeism was assessed using the following question: ‘The last time you experienced depression, how many working days did you have to take off work because of your depression’? Data collected from individuals on their reported salary was used to convert the measures of absenteeism and presenteeism into US dollar purchasing parities based on a conversion factor from the World Bank [15] to estimate the cost associated with depression in the workplace using a human capital approach.

### Statistical analysis

Individual and country characteristics are presented for each country. A high proportion of participants had zero costs associated with presenteeism/absenteeism, and thus, the data followed skewed distributions. We, therefore, used a modified Park test [16] to select the most appropriate distribution. Parameter estimates suggested a Gaussian distribution had the best fit for presenteeism costs, while a Poisson distribution had the best fit for absenteeism costs. Consequently, two generalized linear models were used to examine bivariate and multivariable factors associated with: (a) depression-related absenteeism costs and (b) depression-related presenteeism costs. Country contextual variables (i.e., prevalence of employees with a previous diagnosis of depression and per capita GDP) were computed as an average rating for each country across respondents, and each variable was standardized (i.e., z-score was computed). Post-stratification survey weights, based on gender, age and region of residence, which were aligned with nationally representative figures, were used in all analyses. We used generalized estimating equations (GEE) with robust variance estimates to model within-country correlations [17]. We selected GEE instead of mixed regression models as we were interested in understanding the influence of overall cultural factors rather than individual country-level effects. As GEE is a non-likelihood-based method, Pan’s QIC was used for variable selection and to select the working correlation matrix [18]. Given the diversity in country economic circumstances, we also investigated whether the relationship between fear of losing one’s job and productivity (absenteeism and presenteeism) differed by country GDP, testing the interaction between these variables. All analyses were carried out using SAS version 9.3 and Stata version 11.

### Ethics statement

This study was classified as exempt by the King’s College London, Psychiatry, Nursing, and Midwifery Research Ethics Subcommittee as this was secondary data and was fully anonymized. Data collection was performed independently by Ipsos MORI in accordance with the standards of ESOMAR, AIMRI, and EFAMRO in Europe, and is in line with the data protection act 1998. Data were collected as part of a market research survey and are hosted with the market research agency Ipsos MORI. All data for the market research survey are anonymous and did not include any personal information. No minors or children were involved in the study, and written consent was obtained.

## Results

### Participant characteristics and country averages

Individual sociodemographic characteristics and weighted country averages for mental health and employment characteristics are described in Table 1. As expected, given the diversity of countries included in the sample, there was some variation between countries in relation to education and income.

**Table 1:** Characteristics of employee respondents in each of the participating countries (weighted percent, 95 % confidence interval)

	Brazil (*n* = 1000)	Canada (*n* = 1000)	China (*n* = 1000)	Japan (*n* = 1000)	South Korea (*n* = 1000)	Mexico (*n* = 1000)	South Africa (*n* = 1061)	USA (*n* = 1000)
Gender								
Male	57.3 (54.1, 60.4)	51.9 (48.8, 55.0)	55.1 (51.8, 41.6)	56.5 (53.4, 59.5)	58.6 (55.5, 61,6)	65.8 (62.8, 68.9)	40.0 (36.9, 43.1)	52.5 (49.4, 55.7)
Female	42.7 (39.6, 45.9)	48.1 (45.0, 51.2)	44.9 (41.6, 48.2)	43.5 (40.5, 46.6)	41.4 (38.4, 44.5)	34,2 (31.1, 37.2)	60.0 (56.9, 63.1)	47.5 (44.3, 50.6)
Age								
18–24	32.7 (29.7, 35.7)	26.0 (23.3, 28.8)	27.6 (24.8, 30.5)	17.5 (15.1, 19.8)	15.5 (13.3, 17.7)	31.9 (28.6, 35.2)	40.8 (37.5, 44.1)	20.4 (17.9, 22.8)
25–44	40.1 (37.0, 43.2)	35.3 (32.3, 38.2)	39.8 (36.7, 42.9)	38.6 (35.5, 41.6)	40.8 (37.7, 43.8)	41.7 (38.3, 45.1)	60.0 (56.8, 63.2)	37.5 (34.5, 40.6)
45–64	27.2 (24.4, 30.0)	38.7 (35.7, 41.7)	32.6 (29.1, 36.1)	44.0 (40.9, 47.1)	43.7 (40.6, 46.8)	26.4 (23.4, 29.4)	19.0 (16.8, 21.2)	42.1 (38.9, 45.2)
Education								
No formal qualification	49.9 (44.5, 55.4)	3.3 (2.0, 4.8)	0.2 (0, 0.5)	0.2 (0, 0.5)	17.6 (15.2, 19.9)	76.0 (69.7, 82.3)	4.3 (3.0, 5.7)	1.6 (0.7, 2.7)
Educational title <University	45.2 (40.0, 50.6)	55.6 (48.4, 62.8)	40.8 (34.5, 47.4)	52.4 (46.0, 55.2)	19.8 (17.3, 22.3)	23.9 (19.4, 28.3)	57.1 (49.5, 64.5)	36.7 (29.9, 43.4)
University or above	4.8 (2.7, 6.9)	34.6 (30.1, 39.0)	58.9 (54.0, 63.8)	47.3 (42.8, 51.9)	62.7 (59.6, 69.7)	0.2 (0, 0.5)	38.8 (33.9, 43.6)	49.2 (42.6, 55.7)
Annual income in								
USD	19700	55000	7844	51462	25393	12599	17328	55000
Median (IQR)	(12313, 30782)	(24387, 75377)	(4902, 9805)	(32749, 70175)	(19750, 36678)	(11887, 13334)	(9206, 22076)	(40000, 87500)
Previous diagnosis of depression	18.8 (16.3, 21.3)	20.7 (18.2, 23.2)	6.4 (4.8, 8.1)	10.0 (8.1, 11.9)	7.4 (5.8, 9.0)	14.6 (12.3, 17.0)	25.6 (22.9, 28.4)	22.7 (20.0, 25.3)
Didn’t tell employer about depression because fear of losing job/economic climate	2.4 (0.1, 4.8)	7.3 (3.7, 10.9)	6.6 (0.1, 13.2)	12.0 (5.5., 18.5)	8.0 (1.7, 14.3)	3.0 (0.3, 5.8)	7.2 (3.8, 10.5)	11.4 (7.2, 15.7)
Number of days taken off during episode of depression								
0	65.4 (58.3, 72.5)	42.1 (35.0, 49.2)	29.6 (16.3, 42.8)	23.1 (15.3, 32.8)	67.4 (56.1, 78.6)	65.6 (57.5, 73.8)	49.0 (42.8, 55.3)	58.6 (51.9, 65.3)
1–5	3.0 (0.5, 5.5)	12.6 (7.8, 17.3)	35.0 (22.4, 47.7)	9.0 (3.4, 15.3)	18.3 (9.1, 27.6)	23.8 (16.3, 31.3)	20.5 (15.4, 25.6)	19.9 (14.4, 25.4)
6–10	1.8 (0.0, 3.9)	2.1 (0.1, 4.2)	15.7 (6.2, 25.1)	10.0 (4.2, 16.6)	2.8 (0.0, 6.8)	2.7 (0.2, 5.1)	9.0 (5.7, 12.3)	5.3 (2.3, 8.2)
11–15	6.6 (2.9, 10.3)	2.8 (0.4, 5.2)	6.5 (0.7, 12.3)	2.9 (0.0, 6.5)	0.0 (0.0, 0.0)	2.6 (0.0, 5.3)	7.6 (4.3, 10.9)	0.4 (0.0, 1.2)
16–20	0.0 (0.0, 0.0)	1.7 (0.0, 3.5)	7.5 (0.0, 16.8)	4.1 (0.1, 8.4)	0.0 (0.0, 0.0)	0.4 (0.0, 1.2)	0.0 (0.0, 0.0)	0.0 (0.0, 0.0)
21+	17.7 (12.0, 23.4)	19.5 (13.9, 25.2)	5.8 (0.1, 11.6)	21.8 (14.3, 31.3)	2.8 (0.0, 6.8)	2.3 (0.1, 4.5)	5.9 (3.3, 8.5)	3.7 (1.3, 6.2)
Don’t know	5.4 (2.0, 8.9)	19.2 (13.6, 24.9)	0.0 (0.0, 0.0)	25.1 (17.2, 35.1)	8.6 (1.9, 15.4)	2.7 (0.3, 5.2)	7.9 (4.5, 11.4)	12.1 (7.6, 16.7)
Working status								
Full time’	79.6 (73.6, 85.7)	52.9 (45.7, 60.0)	93.2 (86.5, 99.9)	65.0 (55.2, 74.8)	66.9 (55.5, 78.2)	63.3 (55.1, 71.4)	67.8 (61.8, 73.7)	67.7 (61.3, 74.1)
Part time’	17.2 (11.6, 22.9)	38.6 (31.6, 45.6)	6.8 (0.1, 13.5)	29.7 (20.3, 39.0)	25.8 (15.3, 36.3)	36.7 (28.6, 45.0)	22.8 (17.5, 28.1	26.9 (20.8, 32.9)
Previously employed in the last 12 months	3.1 (0.4, 5.9)	8.5 (4.5, 12.6)	0 (0.0, 0.0)	5.3 (0.7, 10.0)	5.1 (1.0, 13.7)	0 (0.0, 00)	9.4 (5.5, 13.4)	5.4 (2.4, 8.4)

Less than 10 % of respondents in China (6.4 %) and South Korea (7.4 %) reported having a previous diagnosis of depression by a doctor or medical professional, while more than 20 % reported a previous diagnosis in Canada (20.7 %), USA (22.7 %) and South Africa (25.6 %). There was substantial inter-country variation in number of days off, with sample proportions reporting 21+ days off work due to their depression varying from 2.3 % in Mexico to 21.8 % in Japan. Respondents in Japan and the US were the most likely to report not telling their employer about their depression because of fear of losing their job or due to the economic climate (12.0 and 11.4 %, respectively), in contrast to fewer than 5 % in Brazil and Mexico.

### Productivity costs of depression associated with absenteeism and presenteeism across countries

Mean annual per person costs for absenteeism associated with depression were lowest in South Korea at $181. Although Japan had a relatively low prevalence of employees who reported a diagnosis of depression, the average cost of absenteeism associated with depression was highest in Japan ($2674) as a high number of employees took time off of work for at least 10 days. Japan also had the highest aggregate costs of absenteeism associated with depression (almost $6 billion), when considering the size of the labor force in the country and the estimated annual prevalence of depression among employed persons. To account for differences in, for example, salary levels across countries, we also expressed the aggregate costs as a proportion of country GDP. The proportion was highest in Brazil and South Africa (0.7 %) and lowest in South Korea (0.01 %) (see Table 2).

**Table 2:** Annualized population level estimates of productivity costs of depression associated with absenteeism by country, measured in USD

	Brazil	Canada	China^a^	Japan	Korea	Mexico	South Africa	USA
Mean cost/person	1,361	1,567	136	2,674	181	928	894	**390**
Median cost/person	0	0	70	1769	0	0	0	**0**
IQR/person	0, 1,176	0, 1,742	0, 254	0, 4954	0, 15	0, 561	0, 318	**0, 307**
Size of labor force^b^	104,745,358	19,271,114	787,632,272	65,281,090	25,765,461	52,847,521	19,083,339	158,666,072
Estimated annual prevalence employees with diagnosis of depression^c^	10.44	8.28	3.76	3.33	2.96	7.30	12.80	9.66
Aggregate cost (total labor force)	14,889,436,256	2,500,380,791	4,032,677,233	5,818,721,155	138,041,034	3,580,102,463	2,183,744,648	5,977,322,278
% GDP	0.66	0.14	0.04	0.12	0.01	0.28	0.62	0.04

Bold indicates *p* < 0.05

^a^Estimate based on individual rather than household income for China only

^b^Size of the labor force was taken from the International Labor Organization, Key Indicators of the Labor Market Database (2009–2013)

^c^As only lifetime diagnosis of depression was collected, we divided the prevalence estimates collected in this study (as shown in Table 1) by the ratio of lifetime to annual prevalence rates identified for each country as identified by nationally representative estimates from the World Mental Health Survey [46] or national epidemiological surveys [47, 48]

Mean presenteeism cost per person associated with depression was lowest in China at $547; however, it is likely an underestimate relative to the other countries as it is based on individual income rather than household income as is done for the other countries. The USA ($5524) and Brazil ($5788) had the highest presenteeism costs per person associated with depression. Costs of presenteeism associated with depression tended to be 5–10 times higher than those for absenteeism. When taking into account the size of the labor force and the estimated annual prevalence of depression among employed persons, the US was the highest at more than $84 billion and Brazil second at over $63 billion. In terms of proportion of GDP; however, presenteeism costs associated with depression accounted for the greatest proportion in South Africa (4.2 %) and the lowest in Korea (0.1 %). Interestingly, the ratio of presenteeism costs to absenteeism costs varied across countries—being more equal in Japan (1.4) and Canada (2.7), whereas presenteeism accounted for much greater proportions of costs in the US (14.2) and South Africa (6.8) (see Table 3).

**Table 3:** Annualized population level estimates of productivity costs of depression associated with presenteeism by country, measured in USD

	Brazil	Canada	China^a^	Japan	Korea	Mexico	South Africa	USA
Mean cost/person	5,788	4,270	547	3,801	2,114	2,918	6,066	5,524
Median cost/person	4,923	3,011	525	3,639	1,715	2,488	1,300	4,044
IQR	2,532, 7,877	1,994, 5,865	326, 735	1,213, 5,822	821, 2,716	2,466, 3,371	516, 10,187	2,316, 7,414
Size of labor force^b^	104,745,358	19,271,114	787,632,272	65,281,090	25,765,461	52,847,521	19,083,339	158,666,072
Estimated annual prevalence employees with diagnosis of depression^c^	10.44	8.28	3.76	3.33	2.96	7.30	12.80	9.66
Aggregate cost (total labor force)	63,321,129,353	6,813,417,981	16,219,665,046	8,271,114,103	1,612,258,263	11,257,261,838	14,817,220,400	84,663,405,809
% GDP	2.82	0.37	0.18	0.17	0.12	0.89	4.23	0.50

^a^Unemployment rates were taken from the International Labor Organization http://www.ilo.org/global/research/global-reports/global-employment-trends/2014/WCMS_233936/lang–en/index.htm

^b^GDP taken from the World Bank: http://data.worldbank.org/indicator/NY.GDP.PCAP.CD

^c^Though duration and number of episodes may differ by country (e.g., access to appropriate care and treatment). We assumed an average of 37.7 weeks for an episode of depression based on the global burden of disease review and estimate [26]

### Factors associated with absenteeism

When adjusting for all covariates, individuals of middle age (relative to younger age), those with higher levels of education and those with higher incomes tended to have lower levels of depression-related absenteeism. There was a marginal trend for the interaction term for GDP per capita by non-disclosure due to fear of losing one’s job (*p* = 0.08), suggesting that individuals living in countries with higher GDP per capita who did not tell their employer because they feared losing their job were more likely to have higher levels of absenteeism. We repeated the analyses excluding China (due to the difference in income measurement), and the results did not change significantly (see Table 4).

**Table 4:** Factors associated with higher employee absenteeism among individuals with a diagnosis of depression

	Unadjusted		Adjusted		Adjusted with interaction	
	Estimate (95% CI)	*p* value	Estimate (95 % CI)	*p* value	Estimate (95 % CI)	*p* value
Gender						
Female	**0.77 (0.63, 0.92)**	**0.007**	0.94 (0.83, 1.05)	0.26	0.91 (0.82, 1.04)	0.17
Male (ref)	–				–	
Age						
45–64	**0.75 (0.57, 0.97)**	**0.03**	0.97 (0.80,1.19)	0.78	0.96 (0.79, 1.16)	0.68
25–44	**0.65 (0.50, 0.84)**	**0.001**	**0.84 (0.70, 0.99)**	**0.04**	**0.82 (0.68, 0.98)**	**0.03**
18–24	**–**	**–**	**–**	**–**	**–**	**–**
Education						
High	0.72 (0.50, 1.05)	0.09	**0.73 (0.54, 0.99)**	**0.04**	**0.71 (0.52, 0.97)**	**0.03**
Medium	**0.74 (0.58, 0.94)**	**0.02**	**0.85 (0.74, 0.97)**	**0.02**	**0.84 (0.73, 0.96)**	**0.01**
Low	**–**	**–**	**–**	**–**	**–**	**–**
Income						
High	**0.74 (0.66, 0.84)**	**<0.0001**	**0.83 (0.68, 1.00)**	**0.05**	**0.82 (0.67,1.00)**	**0.05**
Medium	**0.84 (0.73, 0.97)**	**0.02**	0.96 (0.83, 1.13**)**	0.62	0.98 (0.84, 1.13)	0.74
Low	**–**	**–**	**–**	**–**	**–**	**–**
Did not tell employer because fear of losing job/economic climate	**1.44 (1.17, 1.78)**	**0.0007**	1.08 (0.79, 1.49)	0.61	0.98 (0.68, 1.43)	0.93
Country prevalence of employees with a diagnosis of depression	0.88 (0.73, 1.07)	0.20	0.90 (0.76, 1.08)	0.26	0.91 (0.75, 1.09)	0.30
GDP per capita	1.13 (0.96, 1.32)	0.14	1.11 (0.94, 1.31)	0.24	1.07 (0.88, 1.31)	0.50
GDP per capita*fear job					1.44 (0.95, 2.20)	0.08

Bold indicates *p* < 0.05

### Factors associated with presenteeism

After adjusting for covariates, individuals with higher levels of education and individuals who did not tell their employer, because they feared losing their job tended to have lower depression-related presenteeism. Individuals with higher incomes had higher depression-related presenteeism. Individuals living in a country with higher prevalence of depression also tended to have higher presenteeism. There was a significant interaction for GDP per capita by non-disclosure due to fear of losing one’s job (*p* < 0.08) suggesting that individuals living in countries with a higher GDP per capita who did not tell their employer because they feared losing their job had higher levels of presenteeism (*p* = 0.0002). As with absenteeism, we repeated the analyses excluding China, and the results did not change significantly (see Table 5).

**Table 5:** Factors associated with higher employee presenteeism among individuals with a diagnosis of depression

	Unadjusted		Adjusted		Adjusted with interaction	
	Estimate (95 % CI)	*p* value	Estimate (95 % CI)	*p* value	Estimate (95 % CI)	*p* value
Gender						
Female	1.19 (0.98, 1.43)	0.07	0.99 (0.96, 1.03)	0.55	0.99 (0.96, 1.03)	0.55
Male (ref)	–		–	–	–	–
Age						
45–64	4.90 (3.32, 7.31)	**<0.0001**	1.03 (0.96, 1.11)	0.43	1.02 (0.95 ,1.09)	0.55
25–44	4.48 (3.00, 3.32)	**<0.0001**	0.96 (0.90, 1.03)	0.26	0.95 (0.90, 1.02)	0.17
18–24	–	–	–	–	–	–
Education						
High	**0.82 (0.75, 0.90)**	**<0.0001**	**0.90 (0.88, 0.93)**	**<0.0001**	**0.90 (0.88, 0.93)**	**<0.0001**
Medium	**0.82 (0.70, 0.95**)	**0.008**	**0.95 (0.91, 0.99)**	**0.02**	**0.96 (0.92, 0.99)**	0.03
Low	–	–	–	–	–	–
Income						
High	**1.25 (1.16, 1.32)**	**<0.0001**	**1.04 (1.01, 1.08)**	**0.03**	**1.04 (1.01, 1.08)**	**0.03**
Medium	**1.77 (1.51, 2.10)**	**<0.0001**	**1.10 (1.08, 1.13)**	**<0.0001**	**1.10 (1.08, 1.13)**	**<0.0001**
Low	–	–	–	–	–	
Did not tell employer because fear of losing job/economic climate	**0.06 (0.01, 0.64)**	**0.02**	**0.80 (0.77, 0.84)**	**<0.0001**	**0.79 (0.75, 0.84)**	**<0.0001**
Country prevalence of employees with a diagnosis of depression	0.97 (0.90, 1.04)	0.37	**1.05 (1.00, 1.10)**	**0.05**	**1.05 (1.01, 1.10)**	**0.05**
GDP per capita	**1.09 (1.02, 1.16)**	**0.01**	0.99 (0.97, 1.02)	0.84	0.99 (0.96, 1.02)	0.48
GDP per capita* fearjob					**1.12 (1.06, 1.20)**	**0.0002**

Bold indicates *p* < 0.05

Though duration and number of episodes may differ by country (e.g., access to appropriate care and treatment). We assumed an average of 37.7 weeks for an episode of depression based on the global burden of disease review and estimate [26]

^a^Unemployment rates were taken from the International Labor Organization http://www.ilo.org/global/research/global-reports/global-employment-trends/2014/WCMS_233936/lang--en/index.htm

^b^GDP taken from the World Bank: http://data.worldbank.org/indicator/NY.GDP.PCAP.CD

## Discussion

To the best of our knowledge, this is the first study to examine the impact of depression on workplace productivity across a diverse set of countries, in terms of both culture and GDP. Previous research on the economic case for tackling depression in the workplace is mainly relevant for Western countries and high-income countries. These findings suggest the impact of depression in the workplace is considerable across all countries, both in absolute monetary terms and in relation to proportion of country GDP. In other words, depression is an issue deserving attention, regardless of a country’s economic development, national income or culture [19–21]. Moreover, with the growth in non-communicable diseases globally—with mental illnesses contributing substantially—the scale of the problem is likely to increase (Bloom et al. [22]).

Although the impact of depression on workplace productivity is universal, there were significant inter-country differences in terms of the prevalence of employees with depression taking time off work, number of days taken off, level of presenteeism and ratio of presenteeism to absenteeism. Most previous studies have been conducted in western or high-income countries, and thus, this study provided an opportunity to explore global similarities and differences. Our study provides higher estimates of work productivity costs compared with previous US studies [8, 23, 24]; however, these studies were based on samples collected more than a decade ago, and there were some methodological differences. We found lower overall productivity costs (in relation to proportion of GDP) associated with depression in Asian countries compared to the US. One driver of lower costs was the lower prevalence of employees diagnosed with depression in Asian countries. In line with previous epidemiological research [25, 26], Asian countries had the lowest prevalence of diagnosis of depression and this may be due to a true difference and/or measurement bias. In the case of the present study, differences could also be due to lower diagnostic rates or a cultural reluctance to disclose depression. Previous research from Japan found a significant relationship between depression (as identified by a psychiatric epidemiological survey using the WHO Composite International Diagnostic Interview [27]) and lower presenteeism, but did not identify a significant relationship between presence of depression and absenteeism [9]. It may be that our sample identified a relationship between depression and absenteeism in Japan as our criteria for depression identified individuals with more severe depression, given they had to receive a diagnosis by a medical professional (Brown et al. [28]; Bebbington et al. [29]) and that there is a high threshold of depression severity which warrants absenteeism in Japan.

We found that presenteeism rates varied according to country characteristics. Individuals living in a country with a higher prevalence of depression diagnoses had higher levels of presenteeism. It may be that prevalence of depression diagnoses also reflects comfort in seeking treatment and or disclosing one’s diagnosis. Previous research has shown that a cultural context which is more open and accepting of mental illness is associated with higher rates of help-seeking, antidepressant use and empowerment, and lower rates of self-stigma and suicide among people with mental illness (Evans-Lacko et al. [30]; Schomerus et al. [31]; Lewer et al. [32]). We also know that openness and support by managers in the workplace are associated with more social acceptance for employees with depression [33]. Thus, it seems that sociocultural and workplace attitudes which promote acceptance and openness about depression could also be important for improving workplace productivity of employees with depression; further research is needed to understand whether this may be at least partially mediated by increased treatment and help-seeking.

Differences in absenteeism and presenteeism were related to economic climate and per capita GDP. Greater reluctance to disclose one’s depression to an employer due to a fear of losing one’s job was related to lower levels of presenteeism. For both absenteeism and presenteeism, this seemed to depend on per capita GDP, in that individuals living in countries with higher per capita GDP who did not disclose their depression to their employer, because they feared losing their job, had higher levels of presenteeism and absenteeism; however, this only reached the level of a trend for absenteeism. Thus, in higher income countries, individuals with depression who experience added stress due to the economic climate may cope through taking time off of work, as this might be more acceptable when the economy is stable, as there is likely to be a stronger social safety net. On the other hand, in lower income countries, individuals who fear disclosing their depression because they may lose their job do not feel comfortable taking time off of work. Consequently, they may remain at work, but have lower levels of productivity, and this is reflected in their relatively lower levels of presenteeism. Some variation may also be due to the fact that the probability of people with depression being employed varies by country and we do not know about differences in the experiences or rates of unemployed people with depression across countries. There is a paucity of data on unemployment rates of depressed persons, though we know that people with mental illness are at a considerable employment disadvantage; for example, in OECD countries, there is a difference in unemployment rate of around 30 percentage points for those with a severe mental disorder and 10–15 percentage points for those with a moderate disorder, when compared to those with no disorder [34]. We also know that adverse labor market conditions and stigmatizing attitudes have a disproportionately negative impact on employment of individuals with mental illness [35]. This difference may be even greater in lower and middle income countries [36].

We also found that absenteeism and presenteeism were associated with individuals’ characteristics. Higher income and education were associated with lower levels of absenteeism. This is supported by previous research, including a large European survey of employed individuals [33] and a meta-analysis of work strain which showed that individuals with higher status occupations had lower levels of absenteeism, and this may be due to their greater financial and interpersonal resources to deal with adverse circumstances [37]. Interestingly, our analyses showed that higher levels of income were associated with higher levels of presenteeism, which would be in line with the importance of financial support. Higher levels of education, however, were associated with lower levels of presenteeism. It is possible that individuals with higher levels of education have a more cognitively demanding job and, therefore, may feel more severely impacted by the cognitive impairments associated with depression (Schultz [38]). Some research has shown that among employees with depression, presenteeism was lower among individuals with jobs involving strong judgement and communication skills [39].

### Strengths and limitations

To the best of our knowledge, this is the first study to examine workplace productivity associated with depression across a diverse range of countries using a common methodology. Our findings come from a unique data set including employees and managers from eight countries, with information on their personal experiences and perceptions of depression in the workplace. Nevertheless, there are several limitations. Diagnosis of depression was based on self-report, and we were not able to control for clinical characteristics, such as severity and/or type of symptoms, and response rates were relatively low. However, the characteristics of respondents are in line with other epidemiological research, as study respondents reporting a diagnosis of depression were more likely to be female, divorced and working part-time. In addition, prevalence of depression diagnosis was lowest in Asian countries. In addition, as the survey only asked about lifetime experience of depression, we had to derive annual prevalence rates from secondary sources. We used estimates from nationally representative psychiatric epidemiology surveys available for each country.

We used the human capital approach to estimate productivity costs, which is still the most commonly used approach across health economics; however, it assumes a societal perspective, and therefore, the associated costs are higher than when using other methods such as friction costs calculations [40, 41]. National mental health policies, employment assistance programs available in the workplace and other policies could be important factors which help explain relationships between depression and productivity in the workplace, and it is a limitation that we have not included this information in our analyses; however, this was beyond the scope of this paper. Additional limitations are that data from this study did not include information on variables such as functioning and work roles, or number and duration of depressive episodes, all of which might be related to workplace productivity.

## Conclusion

Previous research has noted the significant impact of depression on workplace productivity. Our study highlights the individual and country contextual characteristics which influence absenteeism and presenteeism among employees with depression. The trends toward escalating rates of chronic diseases alongside growing economic pressures are an increasing challenge for governments and employers worldwide [42, 43]. There is some evidence of growing interest in improving workplace mental health and an increase in workplace health promotion programs; yet, still only a minority of companies participates in these programs and rates are much lower in low and middle income countries compared to high-income countries [44]. There are a few interventions which have been shown to be cost-effective for addressing depression in the workplace [45], but almost all the available evidence comes from Western, high-income countries. Interventions which support employees with depression need to be developed, adapted, implemented, and evaluated across all countries to mitigate the high personal and societal impacts and economic costs of depression in the workplace.

## Appendix 1: Sensitivity analysis for annualized population level estimates of productivity costs based on range of estimates for the ratio of lifetime prevalence to annual prevalence rates from 1.7 to 3.0

See Tables 6, 7, 8, 9.

**Table 6:** Annualized population level estimates of productivity costs of depression associated with absenteeism by country, measured in USD (applying upper end estimates of the ratio between lifetime prevalence and annual prevalence to be 3.0)

	Brazil	Canada	China^a^	Japan	Korea	Mexico	South Africa	USA
Mean cost/person	1,361	1,567	136	2,674	181	928	894	**390**
Median cost/person	0	0	70	1769	0	0	0	**0**
IQR/person	0, 1,176	0, 1,742	0, 254	0, 4954	0, 15	0, 561	0, 318	**0, 307**
Size of labor force^b^	104,745,358	19,271,114	787,632,272	65,281,090	25,765,461	52,847,521	19,083,339	158,666,072
Estimated annual prevalence employees with diagnosis of depression^c^	6.27	6.90	2.13	3.33	2.47	4.87	8.53	7.57
Aggregate cost (total labor force)	10,720,394,104	2,500,380,791	2,742,220,518	17,456,163,466	138,041,034	2,864,081,970	1,746,995,719	5,618,682,942
% GDP	0.50	0.10	0.03	0.40	0.01	0.20	0.50	0.03

Bold indicates *p* < 0.05

As only lifetime diagnosis of depression was collected, we divided the prevalence estimates collected in this study (as shown in Table 1) by the upper (2.5) and lower (1.7) estimates for the ratio between lifetime prevalence and annual prevalence for countries participating in this study based on estimates from the World Mental Health Survey [47] and national epidemiological surveys [49, 50]

^a^Estimate based on individual rather than household income for China only

^b^Size of the labor force was taken from the International Labor Organization, Key Indicators of the Labor Market Database (2009–2013)

**Table 7:** Annualized population level estimates of productivity costs of depression associated with presenteeism by country, measured in USD (applying upper end estimates of the ratio between lifetime prevalence and annual prevalence to be 3.0)

	Brazil	Canada	China^a^	Japan	Korea	Mexico	South Africa	USA
Mean cost/person	5,788	4,270	547	3,801	2,114	2,918	6,066	5,524
Median cost/person	4,923	3,011	525	3,639	1,715	2,488	1,300	4,044
IQR	2,532, 7,877	1,994, 5,865	326, 735	1,213, 5,822	821, 2,716	2,466, 3,371	516, 10,187	2,316, 7,414
Size of labor force^2^	104,745,358	19,271,114	787,632,272	65,281,090	25,765,461	52,847,521	19,083,339	158,666,072
Estimated annual prevalence employees with diagnosis of depression^**3**^	6.27	6.90	2.13	3.33	2.47	4.87	8.53	7.57
Aggregate cost (total labor force)	45,591,213,134	6,813,417,981	11,029,372,231	9,925,336,924	1,612,258,263	9,005,809,470	11,853,776,320	79,583,601,461
% GDP	2.00	0.40	0.10	0.20	0.10	0.70	3.50	0.50

As only lifetime diagnosis of depression was collected, we divided the prevalence estimates collected in this study (as shown in Table 1) by the upper (2.5) and lower (1.7) estimates for the ratio between lifetime prevalence and annual prevalence for countries participating in this study based on estimates from the World Mental Health Survey [47] and national epidemiological surveys [49, 50]

^a^Estimate based on individual rather than household income for China only

^b^Size of the labor force was taken from the International Labor Organization, Key Indicators of the Labor Market Database (2009–2013)

**Tabel 8:** Annualized population level estimates of productivity costs of depression associated with absenteeism by country, measured in USD (applying lower end estimates of the ratio between lifetime prevalence and annual prevalence to be 1.7)

	Brazil	Canada	China^a^	Japan	Korea	Mexico	South Africa	USA
Mean cost/person	1,361	1,567	136	2,674	181	928	894	**390**
Median cost/person	0	0	70	1769	0	0	0	**0**
IQR/person	0, 1,176	0, 1,742	0, 254	0, 4954	0, 15	0, 561	0, 318	**0, 307**
Size of labor force^b^	104,745,358	19,271,114	787,632,272	65,281,090	25,765,461	52,847,521	19,083,339	158,666,072
Estimated annual prevalence employees with diagnosis of depression^c^	11.06	12.18	3.76	5.88	4.35	8.59	15.06	13.35
Aggregate cost (total labor force)	15,765,285,447	3,677,030,575	4,032,677,233	10,268,331,451	203,001,520	4,211,885,250	2,569,111,351	8,262,769,032
% GDP	0.70	0.20	0.04	0.21	0.02	0.33	0.73	0.05

Bold indicates *p* < 0.05

As only lifetime diagnosis of depression was collected, we divided the prevalence estimates collected in this study (as shown in Table 1) by the upper (2.5) and lower (1.7) estimates for the ratio between lifetime prevalence and annual prevalence for countries participating in this study based on estimates from the World Mental Health Survey [47] and national epidemiological surveys [49, 50]

^a^Estimate based on individual rather than household income for China only

^b^Size of the labor force was taken from the International Labor Organization, Key Indicators of the Labor Market Database (2009–2013)

**Table 9:** Annualized population level estimates of productivity costs of depression associated with presenteeism by country, measured in USD (applying lower end estimates of the ratio between lifetime prevalence and annual prevalence to be 1.7)

	Brazil	Canada	China^a^	Japan	Korea	Mexico	South Africa	USA
Mean cost/person	5,788	4,270	547	3,801	2,114	2,918	6,066	5,524
Median cost/person	4,923	3,011	525	3,639	1,715	2,488	1,300	4,044
IQR	2,532, 7,877	1,994, 5,865	326, 735	1,213, 5,822	821, 2,716	2,466, 3,371	516, 10,187	2,316, 7,414
Size of labor force^b^	104,745,358	19,271,114	787,632,272	65,281,090	25,765,461	52,847,521	19,083,339	158,666,072
Estimated annual prevalence employees with diagnosis of depression^c^	11.06	12.18	3.76	5.88	4.35	8.59	15.06	13.35
Aggregate cost (total labor force)	67,045,901,668	10,019,732,326	16,219,665,046	14,596,083,711	2,370,968,034	13,243,837,457	17,432,024,000	117,034,708,031
% GDP	2.99	0.55	0.18	0.30	0.18	1.05	4.97	0.70

As only lifetime diagnosis of depression was collected, we divided the prevalence estimates collected in this study (as shown in Table 1) by the upper (2.5) and lower (1.7) estimates for the ratio between lifetime prevalence and annual prevalence for countries participating in this study based on estimates from the World Mental Health Survey [47] and national epidemiological surveys [49, 50]

^a^Estimate based on individual rather than household income for China only

^b^Size of the labor force was taken from the International Labor Organization, Key Indicators of the Labor Market Database (2009–2013)
